# Supplementary material for: Size matters but hunger prevails—begging and provisioning rules in blue tit families
Source: PeerJ. 2018 Jul 19;6:e5301. doi: 10.7717/peerj.5301 (PMC6054862; doi:10.7717/peerj.5301)
Supplement: Supplemental Information 2 — All details about data processing, data analyses and raw output are provided. [file peerj-06-5301-s002.docx]

Sizemattersscript.R

Nolwenn

Fri Apr 20 14:03:40 2018

#Package needed
library(phia)

## Loading required package: car

## Warning: package 'car' was built under R version 3.4.4

## Loading required package: carData

## Warning: package 'carData' was built under R version 3.4.4

library(nlme)
library(lme4)

## Warning: package 'lme4' was built under R version 3.4.4

## Loading required package: Matrix

##
## Attaching package: 'lme4'

## The following object is masked from 'package:nlme':
##
## lmList

library(Rmisc)

## Loading required package: lattice

## Loading required package: plyr

library(ggplot2)

#Begging Analysis

#####import data

dataDir <- file.path("C:/Users/Nolwenn/Dropbox/These", "R")
fp<- file.path (dataDir, "begging.csv")
donnees<- read.csv(fp, header=TRUE)
attach(donnees)


##### Average begging and Standard error

dfc <- summarySE(donnees, measurevar="BeggingRaw", groupvars=c("context","Weight"))
dfc

## context Weight N BeggingRaw sd se ci
## 1 none H 42 1.517738 1.895559 0.2924911 0.5906977
## 2 none L 42 3.231667 2.952149 0.4555265 0.9199545
## 3 own H 24 3.680417 4.627079 0.9444985 1.9538440
## 4 own L 26 5.301346 3.813974 0.7479818 1.5404974
## 5 Sibling H 26 3.418462 4.172123 0.8182207 1.6851571
## 6 Sibling L 24 3.513750 3.745995 0.7646480 1.5817950

dfc <- summarySE(donnees, measurevar="BeggingRaw", groupvars=c("Weight"))
dfc

## Weight N BeggingRaw sd se ci
## 1 H 92 2.619076 3.584925 0.3737543 0.7424170
## 2 L 92 3.890163 3.503718 0.3652878 0.7255993

dfc <- summarySE(donnees, measurevar="BeggingRaw", groupvars=c("context"))
dfc

## context N BeggingRaw sd se ci
## 1 none 84 2.374702 2.612136 0.2850074 0.5668682
## 2 own 50 4.523300 4.259148 0.6023345 1.2104365
## 3 Sibling 50 3.464200 3.933181 0.5562358 1.1177976

##### check data
hist(BeggingZ)


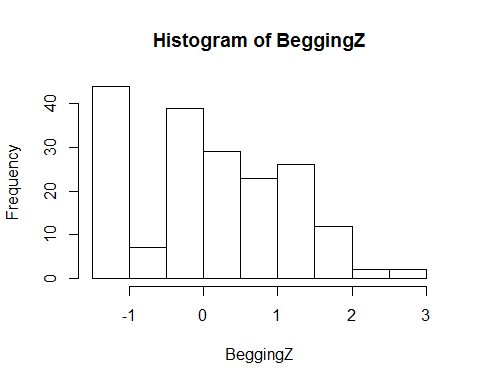


G= ggplot(donnees, aes(x = BeggingZ, color=context))
G + geom_density()


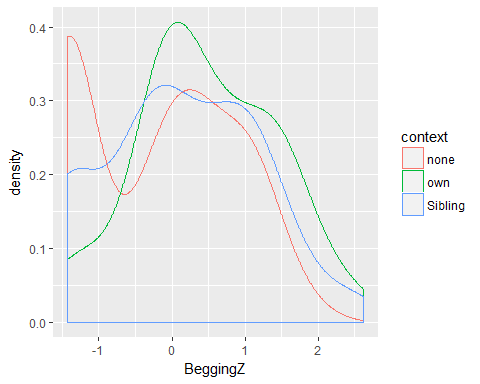


x11()
shapiro.test(BeggingZ)

##
## Shapiro-Wilk normality test
##
## data: BeggingZ
## W = 0.93545, p-value = 2.541e-07

qqnorm(BeggingZ)
qqline(BeggingZ)


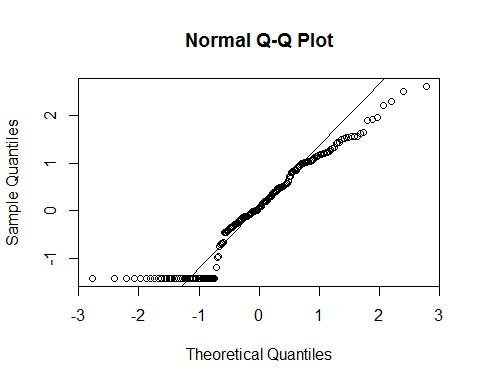


hist(BeggingZ)


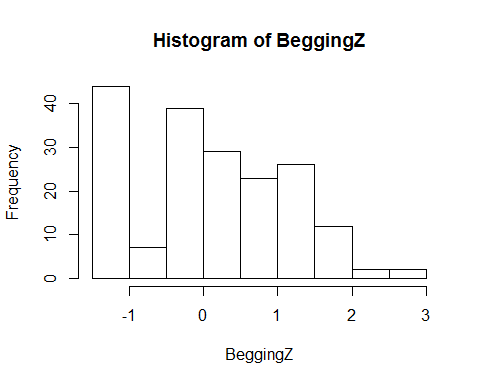


##### Full model begging

lmc<-lmeControl(niterEM=5200,msMaxIter=5200)

M1<- lme(BeggingZ~BroodSize+Weight+ context +
 Weight :context ,
 random=~1|nest/ID, method="REML",
 data=donnees, control=lmc,na.action= na.omit)

summary(M1)

## Linear mixed-effects model fit by REML
## Data: donnees
## AIC BIC logLik
## 525.9234 557.6849 -252.9617
##
## Random effects:
## Formula: ~1 | nest
## (Intercept)
## StdDev: 0.3446617
##
## Formula: ~1 | ID %in% nest
## (Intercept) Residual
## StdDev: 9.645314e-05 0.894707
##
## Fixed effects: BeggingZ ~ BroodSize + Weight + context + Weight:context
## Value Std.Error DF t-value p-value
## (Intercept) 1.4754976 0.6919753 128 2.132298 0.0349
## BroodSize -0.1843784 0.0620044 24 -2.973631 0.0066
## WeightL 0.6256822 0.1952411 25 3.204665 0.0037
## contextown 0.6474490 0.2364261 128 2.738484 0.0071
## contextSibling 0.6256704 0.2343735 128 2.669544 0.0086
## WeightL:contextown 0.0497137 0.3403931 128 0.146048 0.8841
## WeightL:contextSibling -0.5990961 0.3403931 128 -1.760012 0.0808
## Correlation:
## (Intr) BrodSz WeghtL cntxtw cntxtS WghtL:
## BroodSize -0.974
## WeightL -0.141 0.000
## contextown -0.174 0.058 0.413
## contextSibling -0.103 -0.023 0.417 0.298
## WeightL:contextown 0.131 -0.056 -0.574 -0.726 -0.144
## WeightL:contextSibling 0.031 0.056 -0.574 -0.162 -0.720 0.212
##
## Standardized Within-Group Residuals:
## Min Q1 Med Q3 Max
## -2.49026218 -0.70475675 0.04472147 0.67081015 2.46191664
##
## Number of Observations: 184
## Number of Groups:
## nest ID %in% nest
## 26 52

anova(M1)

## numDF denDF F-value p-value
## (Intercept) 1 128 0.609876 0.4363
## BroodSize 1 24 8.791758 0.0067
## Weight 1 25 13.456801 0.0012
## context 2 128 8.547029 0.0003
## Weight:context 2 128 1.689488 0.1887

shapiro.test(resid(M1))

##
## Shapiro-Wilk normality test
##
## data: resid(M1)
## W = 0.99288, p-value = 0.5113

op <- par(mfrow = c(2, 2), mar = c(5, 4, 1, 2))
plot(M1, add.smooth = FALSE, which = 1)


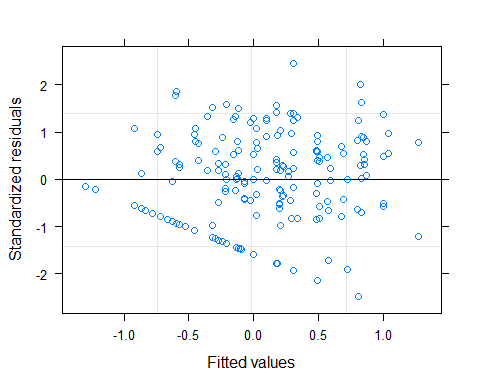


E <- resid(M1)
hist(E, xlab = "Residuals", main = "")
plot(Weight, E, xlab = "Weight ",
 ylab = "Residuals")
plot(context , E, xlab = "context ",
 ylab = "Residuals")
par(op)


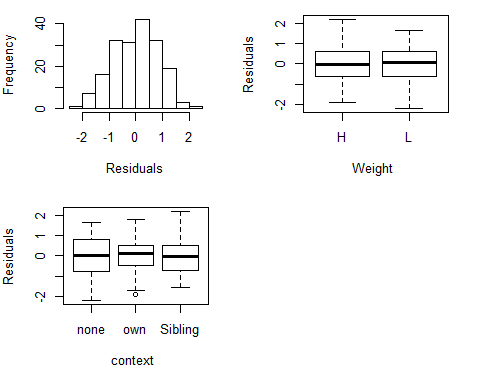


##### Backward selection of the model of begging

M1<- lme(BeggingZ~BroodSize+Weight+ context +
 Weight:context ,
 random=~1|nest/ID, method="ML",
 data=donnees, control=lmc,na.action= na.omit)
summary(M1)

## Linear mixed-effects model fit by maximum likelihood
## Data: donnees
## AIC BIC logLik
## 512.4828 544.6322 -246.2414
##
## Random effects:
## Formula: ~1 | nest
## (Intercept)
## StdDev: 0.3162839
##
## Formula: ~1 | ID %in% nest
## (Intercept) Residual
## StdDev: 9.026685e-05 0.8816981
##
## Fixed effects: BeggingZ ~ BroodSize + Weight + context + Weight:context
## Value Std.Error DF t-value p-value
## (Intercept) 1.4794811 0.6726205 128 2.199578 0.0296
## BroodSize -0.1847251 0.0601716 24 -3.069972 0.0053
## WeightL 0.6256822 0.1961700 25 3.189490 0.0038
## contextown 0.6475654 0.2369318 128 2.733129 0.0072
## contextSibling 0.6247874 0.2343942 128 2.665541 0.0087
## WeightL:contextown 0.0487143 0.3401096 128 0.143231 0.8863
## WeightL:contextSibling -0.5980968 0.3401096 128 -1.758541 0.0810
## Correlation:
## (Intr) BrodSz WeghtL cntxtw cntxtS WghtL:
## BroodSize -0.973
## WeightL -0.146 0.000
## contextown -0.179 0.059 0.414
## contextSibling -0.107 -0.023 0.418 0.305
## WeightL:contextown 0.135 -0.057 -0.577 -0.725 -0.155
## WeightL:contextSibling 0.033 0.057 -0.577 -0.170 -0.718 0.225
##
## Standardized Within-Group Residuals:
## Min Q1 Med Q3 Max
## -2.50051262 -0.70866288 0.05599876 0.65279728 2.51613076
##
## Number of Observations: 184
## Number of Groups:
## nest ID %in% nest
## 26 52

drop1(M1,test= "Chi")

## Single term deletions
##
## Model:
## BeggingZ ~ BroodSize + Weight + context + Weight:context
## Df AIC LRT Pr(>Chi)
## <none> 512.48
## BroodSize 1 518.71 8.2299 0.00412 **
## Weight:context 2 511.98 3.4977 0.17397
## ---
## Signif. codes: 0 '***' 0.001 '**' 0.01 '*' 0.05 '.' 0.1 ' ' 1

M2<- lme(BeggingZ~BroodSize+Weight+ context,
 random=~1|nest/ID, method="ML",
 data=donnees, control=lmc,na.action= na.omit)
summary(M2)

## Linear mixed-effects model fit by maximum likelihood
## Data: donnees
## AIC BIC logLik
## 511.9805 537.7 -247.9903
##
## Random effects:
## Formula: ~1 | nest
## (Intercept)
## StdDev: 0.3233733
##
## Formula: ~1 | ID %in% nest
## (Intercept) Residual
## StdDev: 7.340426e-05 0.8893134
##
## Fixed effects: BeggingZ ~ BroodSize + Weight + context
## Value Std.Error DF t-value p-value
## (Intercept) 1.4690039 0.6726018 130 2.184062 0.0308
## BroodSize -0.1762275 0.0604865 24 -2.913502 0.0076
## WeightL 0.4765026 0.1329986 25 3.582763 0.0014
## contextown 0.6689833 0.1636707 130 4.087374 0.0001
## contextSibling 0.3278203 0.1636707 130 2.002926 0.0473
## Correlation:
## (Intr) BrodSz WeghtL cntxtw
## BroodSize -0.979
## WeightL -0.099 0.000
## contextown -0.118 0.026 -0.016
## contextSibling -0.122 0.026 0.016 0.393
##
## Standardized Within-Group Residuals:
## Min Q1 Med Q3 Max
## -2.34901426 -0.71735324 0.07079209 0.72859600 2.68992608
##
## Number of Observations: 184
## Number of Groups:
## nest ID %in% nest
## 26 52

drop1(M2,test= "Chi")

## Single term deletions
##
## Model:
## BeggingZ ~ BroodSize + Weight + context
## Df AIC LRT Pr(>Chi)
## <none> 511.98
## BroodSize 1 517.43 7.4489 0.0063476 **
## Weight 1 521.93 11.9463 0.0005475 ***
## context 2 524.54 16.5631 0.0002531 ***
## ---
## Signif. codes: 0 '***' 0.001 '**' 0.01 '*' 0.05 '.' 0.1 ' ' 1

M3<- lme(BeggingZ~ Weight+context,
 random=~1|nest/ID, method="ML",
 data=donnees, control=lmc,na.action= na.omit)
summary(M3)

## Linear mixed-effects model fit by maximum likelihood
## Data: donnees
## AIC BIC logLik
## 517.4294 539.934 -251.7147
##
## Random effects:
## Formula: ~1 | nest
## (Intercept)
## StdDev: 0.4234231
##
## Formula: ~1 | ID %in% nest
## (Intercept) Residual
## StdDev: 7.410147e-05 0.8889621
##
## Fixed effects: BeggingZ ~ Weight + context
## Value Std.Error DF t-value p-value
## (Intercept) -0.4427858 0.1481405 130 -2.988959 0.0033
## WeightL 0.4765026 0.1325763 25 3.594176 0.0014
## contextown 0.6773677 0.1640696 130 4.128540 0.0001
## contextSibling 0.3362047 0.1640696 130 2.049159 0.0425
## Correlation:
## (Intr) WeghtL cntxtw
## WeightL -0.447
## contextown -0.427 -0.016
## contextSibling -0.441 0.016 0.399
##
## Standardized Within-Group Residuals:
## Min Q1 Med Q3 Max
## -2.3043002 -0.7343987 0.0433515 0.7026885 2.6003035
##
## Number of Observations: 184
## Number of Groups:
## nest ID %in% nest
## 26 52

drop1(M3,test= "Chi")

## Single term deletions
##
## Model:
## BeggingZ ~ Weight + context
## Df AIC LRT Pr(>Chi)
## <none> 517.43
## Weight 1 527.38 11.951 0.0005463 ***
## context 2 530.24 16.807 0.0002241 ***
## ---
## Signif. codes: 0 '***' 0.001 '**' 0.01 '*' 0.05 '.' 0.1 ' ' 1

M4<- lme(BeggingZ~ context,
 random=~1|nest/ID, method="ML",
 data=donnees, control=lmc,na.action= na.omit)
summary(M4)

## Linear mixed-effects model fit by maximum likelihood
## Data: donnees
## AIC BIC logLik
## 527.38 546.6696 -257.69
##
## Random effects:
## Formula: ~1 | nest
## (Intercept)
## StdDev: 0.3774805
##
## Formula: ~1 | ID %in% nest
## (Intercept) Residual
## StdDev: 0.2573177 0.904302
##
## Fixed effects: BeggingZ ~ context
## Value Std.Error DF t-value p-value
## (Intercept) -0.2052147 0.1328434 130 -1.544787 0.1248
## contextown 0.6771346 0.1698684 130 3.986231 0.0001
## contextSibling 0.3373183 0.1698684 130 1.985763 0.0492
## Correlation:
## (Intr) cntxtw
## contextown -0.480
## contextSibling -0.480 0.341
##
## Standardized Within-Group Residuals:
## Min Q1 Med Q3 Max
## -2.09738388 -0.73955161 -0.04171397 0.74808705 2.38312702
##
## Number of Observations: 184
## Number of Groups:
## nest ID %in% nest
## 26 52

drop1(M4,test= "Chi")

## Single term deletions
##
## Model:
## BeggingZ ~ context
## Df AIC LRT Pr(>Chi)
## <none> 527.38
## context 2 539.26 15.877 0.0003568 ***
## ---
## Signif. codes: 0 '***' 0.001 '**' 0.01 '*' 0.05 '.' 0.1 ' ' 1

##### Full model selected
M2<- lme(BeggingZ~BroodSize+Weight+context,
 random=~1|nest/ID, method="REML",
 data=donnees, control=lmc,na.action= na.omit)
summary(M2)

## Linear mixed-effects model fit by REML
## Data: donnees
## AIC BIC logLik
## 524.616 550.1151 -254.308
##
## Random effects:
## Formula: ~1 | nest
## (Intercept)
## StdDev: 0.3479758
##
## Formula: ~1 | ID %in% nest
## (Intercept) Residual
## StdDev: 9.980121e-05 0.8977733
##
## Fixed effects: BeggingZ ~ BroodSize + Weight + context
## Value Std.Error DF t-value p-value
## (Intercept) 1.4664986 0.6904672 130 2.123922 0.0356
## BroodSize -0.1759498 0.0621630 24 -2.830460 0.0092
## WeightL 0.4765026 0.1324270 25 3.598228 0.0014
## contextown 0.6686378 0.1631850 130 4.097422 0.0001
## contextSibling 0.3274748 0.1631850 130 2.006770 0.0468
## Correlation:
## (Intr) BrodSz WeghtL cntxtw
## BroodSize -0.980
## WeightL -0.096 0.000
## contextown -0.116 0.026 -0.016
## contextSibling -0.119 0.026 0.016 0.394
##
## Standardized Within-Group Residuals:
## Min Q1 Med Q3 Max
## -2.34807656 -0.70162575 0.07289837 0.71572363 2.64684525
##
## Number of Observations: 184
## Number of Groups:
## nest ID %in% nest
## 26 52

anova(M2)

## numDF denDF F-value p-value
## (Intercept) 1 130 0.604215 0.4384
## BroodSize 1 24 8.675335 0.0071
## Weight 1 25 13.365038 0.0012
## context 2 130 8.485332 0.0003

##### Interaction calculation

Mposth<-lme(BeggingZ~BroodSize+context+Weight, random=~1|nest/ID, method="REML", data=donnees)
testInteractions(Mposth, pairwise="context", adjustment="none", label.factors=TRUE)

## Chisq Test:
## P-value adjustment method: none
## Value Df Chisq Pr(>Chisq)
## contextnone-own -0.66864 1 16.7889 4.178e-05 ***
## contextnone-Sibling -0.32747 1 4.0271 0.04477 *
## contextown-Sibling 0.34116 1 3.6071 0.05753 .
## ---
## Signif. codes: 0 '***' 0.001 '**' 0.01 '*' 0.05 '.' 0.1 ' ' 1

#Parental feeding analysis

##### Data import

detach(donnees)
dataDir <- file.path("C:/Users/Nolwenn/Dropbox/These", "R")
fp<- file.path (dataDir, "feeding.csv")
donnees<- read.csv(fp, header=TRUE)
attach(donnees)

##### full model and backward selection

M1<-glmer(cbind(Yes,No) ~ Broodsize + Weight+ context+ Weight:context+( 1| nest/ID),
 family = binomial(cloglog), data = donnees)
summary(M1)

## Generalized linear mixed model fit by maximum likelihood (Laplace
## Approximation) [glmerMod]
## Family: binomial ( cloglog )
## Formula: cbind(Yes, No) ~ Broodsize + Weight + context + Weight:context +
## (1 | nest/ID)
## Data: donnees
##
## AIC BIC logLik deviance df.resid
## 150.1 170.3 -67.1 134.1 84
##
## Scaled residuals:
## Min 1Q Median 3Q Max
## -2.0192 -0.5782 -0.4846 1.0109 3.4485
##
## Random effects:
## Groups Name Variance Std.Dev.
## nest (Intercept) 0 0
## Number of obs: 92, groups: nest, 26
##
## Fixed effects:
## Estimate Std. Error z value Pr(>|z|)
## (Intercept) -1.9577551 1.1681686 -1.676 0.0938 .
## Broodsize 0.0080232 0.0997832 0.080 0.9359
## WeightL 0.0003159 0.5779440 0.001 0.9996
## contextown 1.9671018 0.4875347 4.035 5.47e-05 ***
## contextSibling -1.3700715 1.0804688 -1.268 0.2048
## WeightL:contextown -0.0399509 0.6866308 -0.058 0.9536
## WeightL:contextSibling 1.7884324 1.2399242 1.442 0.1492
## ---
## Signif. codes: 0 '***' 0.001 '**' 0.01 '*' 0.05 '.' 0.1 ' ' 1
##
## Correlation of Fixed Effects:
## (Intr) Brodsz WeghtL cntxtw cntxtS WghtL:
## Broodsize -0.937
## WeightL -0.254 0.007
## contextown -0.371 0.083 0.593
## contxtSblng -0.118 -0.015 0.267 0.316
## WghtL:cntxt 0.293 -0.091 -0.842 -0.713 -0.224
## WghtL:cntxS 0.079 0.039 -0.466 -0.273 -0.872 0.389

drop1(M1,test= "Chi")

## Single term deletions
##
## Model:
## cbind(Yes, No) ~ Broodsize + Weight + context + Weight:context +
## (1 | nest/ID)
## Df AIC LRT Pr(Chi)
## <none> 150.14
## Broodsize 1 148.15 0.0065 0.9359
## Weight:context 2 149.46 3.3202 0.1901

M2<-glmer(cbind(Yes,No) ~ Weight*context+( 1| nest/ID),
 family = binomial(cloglog), data = donnees)
summary(M2)

## Generalized linear mixed model fit by maximum likelihood (Laplace
## Approximation) [glmerMod]
## Family: binomial ( cloglog )
## Formula: cbind(Yes, No) ~ Weight * context + (1 | nest/ID)
## Data: donnees
##
## AIC BIC logLik deviance df.resid
## 148.1 165.8 -67.1 134.1 85
##
## Scaled residuals:
## Min 1Q Median 3Q Max
## -2.0000 -0.5774 -0.5000 1.0000 3.4641
##
## Random effects:
## Groups Name Variance Std.Dev.
## nest (Intercept) 0 0
## Number of obs: 92, groups: nest, 26
##
## Fixed effects:
## Estimate Std. Error z value Pr(>|z|)
## (Intercept) -1.870e+00 4.087e-01 -4.576 4.75e-06 ***
## WeightL -1.298e-10 5.779e-01 0.000 1.000
## contextown 1.964e+00 4.858e-01 4.042 5.29e-05 ***
## contextSibling -1.369e+00 1.080e+00 -1.267 0.205
## WeightL:contextown -3.496e-02 6.838e-01 -0.051 0.959
## WeightL:contextSibling 1.784e+00 1.239e+00 1.440 0.150
## ---
## Signif. codes: 0 '***' 0.001 '**' 0.01 '*' 0.05 '.' 0.1 ' ' 1
##
## Correlation of Fixed Effects:
## (Intr) WeghtL cntxtw cntxtS WghtL:
## WeightL -0.707
## contextown -0.841 0.595
## contxtSblng -0.378 0.267 0.318
## WghtL:cntxt 0.598 -0.845 -0.710 -0.226
## WghtL:cntxS 0.330 -0.466 -0.277 -0.872 0.394

drop1(M2,test= "Chi")

## Single term deletions
##
## Model:
## cbind(Yes, No) ~ Weight * context + (1 | nest/ID)
## Df AIC LRT Pr(Chi)
## <none> 148.15
## Weight:context 2 147.48 3.3269 0.1895

M3<-glmer(cbind(Yes,No) ~ Weight+context+( 1| nest/ID),
 family = binomial(cloglog), data = donnees)
summary(M3)

## Generalized linear mixed model fit by maximum likelihood (Laplace
## Approximation) [glmerMod]
## Family: binomial ( cloglog )
## Formula: cbind(Yes, No) ~ Weight + context + (1 | nest/ID
## Data: donnees
##
## AIC BIC logLik deviance df.resid
## 147.5 160.1 -68.7 137.5 87
##
## Scaled residuals:
## Min 1Q Median 3Q Max
## -2.1005 -0.5742 -0.5472 0.9521 3.6547
##
## Random effects:
## Groups Name Variance Std.Dev.
## nest (Intercept) 0 0
## Number of obs: 92, groups: nest, 26
##
## Fixed effects:
## Estimate Std. Error z value Pr(>|z|)
## (Intercept) -1.9571 0.3290 -5.949 2.70e-09 ***
## WeightL 0.1675 0.2903 0.577 0.564
## contextown 1.9423 0.3419 5.681 1.34e-08 ***
## contextSibling -0.1799 0.5006 -0.359 0.719
## ---
## Signif. codes: 0 '***' 0.001 '**' 0.01 '*' 0.05 '.' 0.1 ' ' 1
##
## Correlation of Fixed Effects:
## (Intr) WeghtL cntxtw
## WeightL -0.478
## contextown -0.736 -0.014
## contxtSblng -0.519 0.025 0.488

drop1(M3,test= "Chi")

## Single term deletions
##
## Model:
## cbind(Yes, No) ~ Weight + context + (1 | nest/ID)
## Df AIC LRT Pr(Chi)
## <none> 147.48
## Weight 1 145.81 0.334 0.5633
## context 2 190.65 47.177 5.698e-11 ***
## ---
## Signif. codes: 0 '***' 0.001 '**' 0.01 '*' 0.05 '.' 0.1 ' ' 1

M4<-glmer(cbind(Yes,No) ~ context+( 1| nest/ID),
 family = binomial(cloglog), data = donnees)
summary(M4)

## Generalized linear mixed model fit by maximum likelihood (Laplace
## Approximation) [glmerMod]
## Family: binomial ( cloglog )
## Formula: cbind(Yes, No) ~ context + (1 | nest/ID)
## Data: donnees
##
## AIC BIC logLik deviance df.resid
## 145.8 155.9 -68.9 137.8 88
##
## Scaled residuals:
## Min 1Q Median 3Q Max
## -1.9704 -0.5774 -0.5222 1.0150 3.8297
##
## Random effects:
## Groups Name Variance Std.Dev.
## nest (Intercept) 4.08e-16 2.02e-08
## Number of obs: 92, groups: nest, 26
##
## Fixed effects:
## Estimate Std. Error z value Pr(>|z|)
## (Intercept) -1.8698 0.2890 -6.471 9.75e-11 ***
## contextown 1.9457 0.3418 5.692 1.26e-08 ***
## contextSibling -0.1872 0.5004 -0.374 0.708
## ---
## Signif. codes: 0 '***' 0.001 '**' 0.01 '*' 0.05 '.' 0.1 ' ' 1
##
## Correlation of Fixed Effects:
## (Intr) cntxtw
## contextown -0.845
## contxtSblng -0.577 0.488

drop1(M4,test= "Chi")

## Single term deletions
##
## Model:
## cbind(Yes, No) ~ context + (1 | nest/ID)
## Df AIC LRT Pr(Chi)
## <none> 145.81
## context 2 189.33 47.522 4.795e-11 ***
## ---
## Signif. codes: 0 '***' 0.001 '**' 0.01 '*' 0.05 '.' 0.1 ' ' 1

##### full model selected

M4<-glmer(cbind(Yes,No) ~ context+( 1| nest/ID),
 family = binomial(cloglog), data = donnees)
summary(M4)

## Generalized linear mixed model fit by maximum likelihood (Laplace
## Approximation) [glmerMod]
## Family: binomial ( cloglog )
## Formula: cbind(Yes, No) ~ context + (1 | nest/ID)
## Data: donnees
##
## AIC BIC logLik deviance df.resid
## 145.8 155.9 -68.9 137.8 88
##
## Scaled residuals:
## Min 1Q Median 3Q Max
## -1.9704 -0.5774 -0.5222 1.0150 3.8297
##
## Random effects:
## Groups Name Variance Std.Dev.
## nest (Intercept) 4.08e-16 2.02e-08
## Number of obs: 92, groups: nest, 26
##
## Fixed effects:
## Estimate Std. Error z value Pr(>|z|)
## (Intercept) -1.8698 0.2890 -6.471 9.75e-11 ***
## contextown 1.9457 0.3418 5.692 1.26e-08 ***
## contextSibling -0.1872 0.5004 -0.374 0.708
## ---
## Signif. codes: 0 '***' 0.001 '**' 0.01 '*' 0.05 '.' 0.1 ' ' 1
##
## Correlation of Fixed Effects:
## (Intr) cntxtw
## contextown -0.845
## contxtSblng -0.577 0.488
